# E2A attenuates tumor-initiating capacity of colorectal cancer cells via the Wnt/beta-catenin pathway

**DOI:** 10.1186/s13046-019-1261-5

**Published:** 2019-06-24

**Authors:** Hongchao Zhao, Chunlin Zhao, Haohao Li, Danhua Zhang, Guanghui Liu

**Affiliations:** Department of General Surgery, The First Affiliated Hospital of Zhengzhou University, 41 Jianshe Road, Zhengzhou, Henan China

**Keywords:** Helix–loop–helix protein e12, Helix-loop-helix protein e47, Transcription factors, Epithelial-mesenchymal transition, FoxM1 protein, Human, Tumor suppressor gene

## Abstract

**Background:**

The E2A gene, which encodes two basic helix–loop–helix transcription factors, E12 and E47, regulates colorectal cancer progression and epithelial-mesenchymal transition. However, whether E2A regulates the tumor-initiating capacity of colorectal cancer is unclear. Thus, we have studied E2A expression in the initiation of colorectal cancer in vivo and in vitro.

**Methods:**

Immunohistochemistry and immunoblot were performed to determine protein levels of E2A in colorectal cancer specimens and cells. RNAi was employed to downregulate E2A expression, and the subsequent change in protein level was evaluated by immunoblot. Sphere-forming assay and enumeration of liver metastasis in mouse models were used to identify the tumor formation ability of colorectal cancer cells.

**Results:**

E2A expression in colorectal cancer clinical specimens was inversely associated with patients’ progression-free survival. Functional studies demonstrated that E2A significantly decreased tumor formation in vitro and in vivo*.* Furthermore, nuclear translocation of beta-catenin and activation of the Wnt/beta-catenin pathway occurred after suppression of E2A in colorectal cancer cells. FoxM1 was identified as a down-stream target by mRNA microarray, implying that FoxM1 plays a main role in determining how E2A regulates the tumor-initiating capacity of colorectal cancer.

**Conclusion:**

E2A suppresses tumor-initiating capacity by targeting the FoxM1-Wnt/β-catenin pathway.

**Electronic supplementary material:**

The online version of this article (10.1186/s13046-019-1261-5) contains supplementary material, which is available to authorized users.

## Introduction

Colorectal cancer (CRC) includes a group of heterogeneous malignant tumors arising in any part of the large intestine, with the capacity to spread to other tissues of the body, mostly to the liver and lung [1]. CRC is one of the most prevalent malignancies leading to cancer-related death worldwide, ranking third in men and women [2]. Most cancer-related deaths are caused by cancer metastasis [3]. The metastasis cascade is a two-phase process: In the first phase, cancer cells in primary tumors break away from the microenvironment and invade via the basement membrane. In the second phase, the cancer cells develop into a metastatic, full-fledged tumor at a distant site [4, 5]. The second phase takes place only if the cancer cells possess a tumor-initiating ability [6]; this ability is not well understood.

Genetic instability spawns distinct clonal subpopulations, which are accompanied by tumor progression, resulting in clonal heterogeneity in tumors [7]. Accumulating evidence points to the existence of a subclass of neoplastic cells within tumors that have the ability to self-renew, resist chemotherapy, and seed as secondary tumors; these cells are termed cancer stem cells [8–11]. The origin of cancer stem cells is controversial, but cancer cells gaining stem-cell properties as they undergo epithelial-mesenchymal transitions (EMT) have been reported [12, 13]. Through EMT, cancer cells with differentiated epithelial phenotypes lose apicobasal polarity, become motile, and express markers characteristic of mesenchymal cells [14–16]. For instance, ectopic expression of beta-catenin, which is strongly correlated with EMT in CRC cells, has caused dedifferentiation of post-mitotic intestinal epithelial cells, leading to the generation of tumor-initiating cells in vivo [17, 18].

E12 and E47, two basic helix–loop–helix (bHLH) transcription factors, are encoded by the E2A gene through variant splicing [19]. E12 and E47, which belong to the class I bHLH family, regulate target genes by binding DNA with tissue-specific Class II HLH proteins, either as homodimers or heterodimers [20–22]. It has been reported [23, 24] that E12/E47 expression was decreased in CRC tissues and inhibited the proliferation and metastasis of CRC cells in vitro*.* In addition, we found that shE2A induced EMT and up-regulated the expression of beta-catenin in CRC cells [24]. The effects of E2A on the tumor-initiating capacity of CRC cells remains unclear.

In the present study, we examined E2A expression in CRC tissues in relation to progression-free survival of CRC patients. Decreased expression of E2A promoted the tumorigenic capacity of CRC cells in vivo and in vitro*.* Functional assays revealed that the canonical Wnt/β-catenin pathway critically affects E2A on CRC cells. Furthermore, we identified FoxM1 as a novel target of E2A and showed that FoxM1 plays a critical role in E2A-regulated inhibition of cancer-initiating capacity.

## Materials and methods

### Cell culture

Human CRC cell lines, SW480 and Caco-2, were purchased from the American Type Culture Collection (Manassas, VA, USA). SW480 was cultured in Leibovitz’s L-15 Medium (Corning Cellgro^®^, Manassas, VA, USA) and Caco-2 in MEM Medium (Corning Cellgro^®^). All culture media were supplemented with 10% fetal bovine serum (Invitrogen, Carlsbad, CA, USA). Cells were maintained at 37 °C/5% CO_2_ in a humidified incubator. Recombinant human Wnt3a (R&D System, Minneapolis, MN, USA) was used at a concentration of 100 ng/mL for treating Caco-2/E12 and Caco-2/E47 cells to activate β-catenin. CGP049090 (Sigma-Aldrich, Lyon, France), a small-molecule inhibitor of Wnt/β-catenin, was diluted in 10 μM for treating SW480/shE2A cells.

### Clinical specimens

The clinical research protocol was approved by the Ethics Committee of The First Affiliated Hospital of Zhengzhou University. Two hundred sixteen surgical specimens of primary CRC tumors were obtained from The First Affiliated Hospital of Zhengzhou University, in 2015–2017, with written informed consent given by all patients before operation. Patients were excluded if they had received neoadjuvant chemoradiotherapy, had unresectable colorectal cancers, had tumors of other organs, or were unlikely to be interviewed during the follow-up. The demographic and clinic-pathological characteristics of all included patients are presented in Table 1. Fresh tumor tissues were harvested immediately after dissection, snap-frozen in liquid nitrogen, and preserved at − 80 °C. Tumors were classified/staged according to the Cancer Staging Manual of the International Union Against Cancer (7th edition, 2009).

**Table 1:** Patients’ Demographic and Clinicopathological Data

CHARACTERISTIC	Number (%) of cases
Age	
40–59	75 (34.7%)
60–79	130 (60.2%)
> 80	11 (5.1%)
Gender	
Male	119 (55.1%)
Female	97 (44.9%)
Tumor histology	
Tubular adenocarcinoma	126 (58.3%)
Mucinous adenocarcinoma	74 (34.3%)
Papillary adenocarcinoma	16 (7.4%)
Tumor site	
Rectum and sigmoid	97 (44.9%)
Right colon	94 (43.5%)
Left colon	25 (11.6%)
Tumor size	
≤ 5 cm	103 (47.7%)
> 5 cm	113 (52.3%)
TNM stage	
I	57 (26.4%)
II	62 (28.7%)
III	80 (37.0%)
IV	17 (7.9%)

Immunohistochemistry staining and scoring of each slide was performed as previously described [23]. Slides were examined by two researchers (Liu and Zhang) independently. Staining intensity was scored as 0 (no staining), 1 (weak staining), 2 (moderate staining), and 3 (strong staining); positive cells on each section were scored as 0 (< 10%), 1 (10–25%), 2 (26–50%), and 3 (> 50%). The final score was a product of the intensity scores and the number of positive cells per slide. Slides with a score of 0–3 were defined as low expression and those with a score of 4–9 were defined as high expression.

### Establishment of stable cell lines and transient transfection

Plasmids pL/shRNA/F-shR with shE2A or shNC (negative control) (Novobio, Shanghai, China) were applied in lentivirus transfection to SW480 cells for establishing shE2A/shNC-expressing stable clones, according to the manufacturer’s instructions. Plasmids, pEZ-M29-E12 and pEZ-M29-E47, encoding fusion proteins eGFP-E12 and eGFP-E47 were purchased from Genecopoeia (Rockville, MD, USA). Short hairpin RNA (shRNA) against human FoxM1 and shRNA-negative control (shNC) were bought from GenePharma (Shanghai, China). Transient transfection was conducted according to established protocols [24]. Levels of the targeted genes were assessed with immunoblot, with glyceraldehyde-3-phosphate dehydrogenase (GAPDH) as the loading control.

### Protein extraction and immunoblot

Cell lysate preparation and immunoblot analysis were performed according to established protocols [25]. A radioimmunoprecipitation buffer (Solarbio, Beijing, China), containing a protease inhibitor cocktail (Roche Applied Science, Basel, Switzerland) and a phosphatase inhibitor cocktail (CST), was used to extract total protein, and the Subcellular Protein Fractionation Kit for Cultured Cells (Thermo-Fisher Scientific; Waltham, MA, USA) was used according to the manufacturer’s protocol. Western blot was conducted according to standard protocol, with primary antibodies of E2A (Santa Cruz, Dallas, Texas, USA), β-catenin (Cell Signaling Technol.), Phospho-β-Catenin (Ser33/37/Thr41) (Cell Signaling Technol.), TCF-1 (Cell Signaling Technol.), Lgr5 (Abcam, Cambridge, UK.), FoxM1 (Cell Signaling Technol.), and cyclin D1 (Cell Signaling Technol.). Goat anti-mouse or goat anti-rabbit IgG conjugated to horseradish peroxidase (HRP; Pierce, Rockford, IL, USA) was used as the secondary antibody. SuperSignal West Pico Chemiluminescent Substrate (Pierce) was used for visualizing chemiluminescent signals; the signal intensity was analyzed with Image Lab™ Software Version 4.0.1 (BIO-RAD, Hercules, CA, USA). The experiments were performed in triplicate, with GAPDH (Kangchen, Shanghai, China) or specificity protein 1 (SP1, Cell Signaling Technology) used as the endogenous control.

### Sphere-forming assay

Cells were harvested with 0.25% trypsin/EDTA and suspended at a concentration of 5 × 10^5^ cells/mL in tryptose sulfite cycloserine medium (minimal essential medium supplemented with 10 ng/mL epidermal growth factor) (Thermo-Fisher Scientific), 10 ng/mL basic fibroblast growth factor (Thermo-Fisher Scientific), 10 ng/mL Noggin (Thermo-Fisher Scientific) and 1000 U/mL leukemia inhibitory factor (Thermo-Fisher Scientific). Cells were cultured in 3.5-cm dishes precoated with poly-hema for 14 d to form colonies.

### Liver metastasis in mouse models

All animal procedures were performed with the approval of the Local Medical Experimental Animal Care Commission of the First Affiliated Hospital of Zhengzhou University. Four-week-old male BALB/c nu/nu mice were purchased from the Chinese Academy of Sciences, Shanghai, and raised in a specific-pathogen-free environment. Six nude mice were included in each group for establishment of the liver metastasis model. Laparotomies on anesthetized mice were performed to expose the spleen, followed by slow injection of 1 × 10^7^ cells into the spleen. The spleen was returned to the abdominal cavity, and the injected cells flowed via the circulatory system to the liver. Six weeks after injection, the mice were euthanized, and hematoxylin-eosin-stained sections of liver were examined microscopically for metastases.

### mRNA microarray

Microarray experiments were performed by KangChen Bio-tech, Shanghai, China. The Agilent Whole Genome Oligo Microarray (Santa Clara, CA, USA) was used to identify mRNA transcripts with differential expression between SW480/shE2A cells and SW480/shNC cells. CRC cell samples were used for the array analysis according to the manufacturer’s protocol in quadruplicate.

### TCF/LEF reporter luciferase assay

TCF/LEF reporter luciferase assay was performed with a TCF/LEF Reporter Kit (BPS Bioscience, San Diego, CA, USA). SW480/shE2A cells treated with/without CGP049090 and SW480/shNC were transfected with pTOPFLASH (Tcf/Lef reporter) and pFOP-FLASH (mutated Tcf/Lef binding sites) for 48 h. Luciferase activity was measured with the Dual Luciferase Reporter Assay (Promega, Madison, WI, USA). Firefly luciferase activity was normalized to *Renilla* luciferase activity for each transfected well. All transfection experiments were conducted in triplicate and repeated three times independently. Data are expressed as the mean ± SD.

### Statistical analysis

A two-tailed Student’s *t*-test, χ^2^ Test, multivariate Cox’s proportional hazards models, and one-way ANOVA were used for statistical analysis as appropriate. The effect of E2A on survival was estimated with the Kaplan-Meier curve and log-rank test. All statistical analyses were performed with SPSS 16.0 (SPSS Inc., Chicago, IL, USA). A two-tailed value of *P* < 0.05 was considered statistically significant.

## Results

### E2A expression correlates with progression-free survival of CRC

**Fig. 1:**
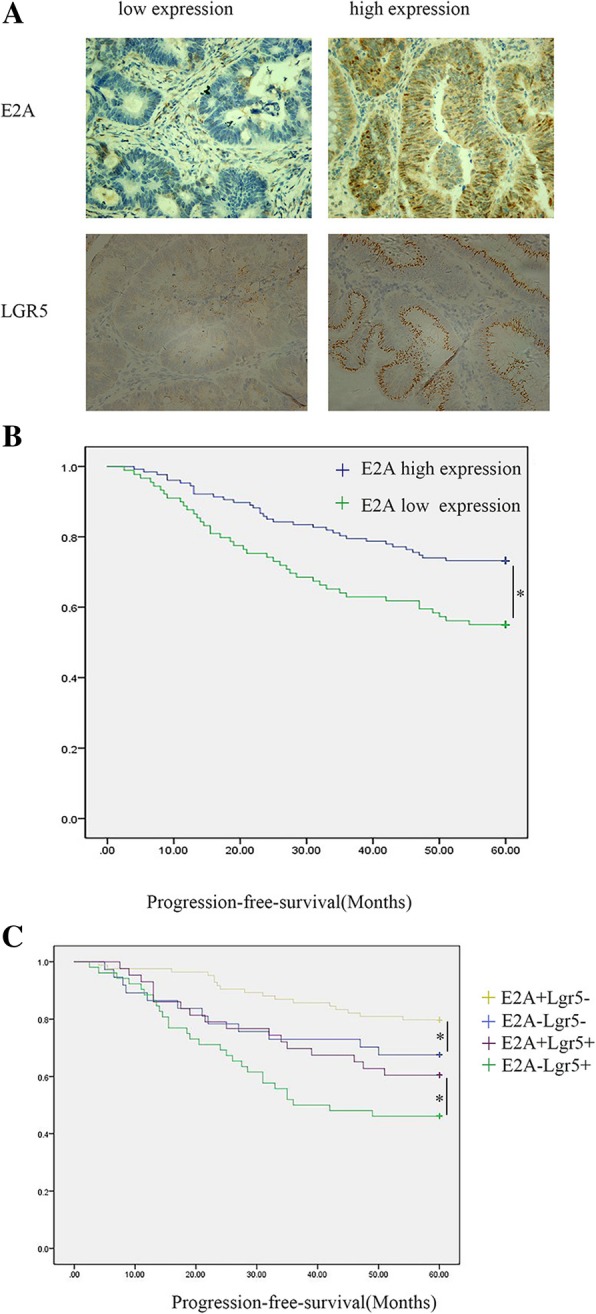
E2A expression correlates with progression-free survival of colorectal cancer. **a** E2A expression (upper panel) and Lgr5 expression (lower panel) in representative immunohistochemistry images. 200×. **b** Progression-free survival of patients with high and low E2A expression (*P* < 0.05). **c** Progression-free survival of patients with respective E2A and Lgr5 expression (*P* < 0.05)

As previously described [22], E2A expression is decreased in CRC tissues. The tumor-initiating capacity of cancer cells induces cell proliferation in CRC after surgery, which leads to tumor recurrence and metastasis. To verify whether expression of E2A is correlated with progression-free survival, we evaluated the expression of E2A protein in 216 CRC tissues with immunohistochemistry staining (Fig. 1a). Using Kaplan-Meier 5-year survival curves, we examined the differences in outcomes between CRC patients with low and high E2A expression. Patients with high E2A expression had longer 5-year progression-free survival than did patients with low expression (Fig. 1b): 73.2% versus 55.1 (*P* < 0.05). We also performed multivariate Cox regression analysis for PFS in CRC patients, which revealed that E2A expression predicted worse PFS (Table 2, OR 1.86, 95%CI 1.17–2.95, *P* = 0.009). Hence, E2A expression seems to be a predictor for progression-free survival in CRC patients.

**Table 2:** Multivariate Cox regression analysis for PFS in CRC patients

PFS	OR	95%CI	*P*
Age	1.001	0.980–1.022	0.947
Gender	1.061	0.648–1.737	0.814
Tumor Histology	1.060	0.746–1.507	0.744
Tumor Size	1.314	0.801–2.153	0.279
Tumor Site	0.637	0.392–1.035	0.068
E2A expression	1.86	1.17–2.95	0.009
TNM stage	4.53	3.131–6.554	0.000

The putative role of Lgr5 as a CRC stem-cell marker [25, 26] has aroused attention. Therefore, we evaluated Lgr5 expression in CRC with immunohistochemistry (Fig. 1a). As illustrated in Table 3, the expression of E2A was inversely associated with Lgr5 expression (*P* < 0.05), which indicates that E2A expression may correlate with tumor-initiating capacity of CRC cells. To study the relationship between E2A, Lgr5 and patients’ progression-free survival, we performed Kaplan-Meier analyses, as illustrated in Fig. 1c. The E2A + Lgr5- group had the longest 5-year progression-free survival, and the E2A-Lgr5+ group had the shortest survival (*P* < 0.05); there was no significant difference in progression-free survival between the E2A-Lgr5- group and E2A + Lgr5+ group (*P* > 0.05).

**Table 3:** Relationship of e2a expression to lgr5 expression

	Lgr5 high expression	Lgr5 low expression	Total
E2A high expression	43 (19.9%)	84 (38.8%)	127 (58.8%)
E2A low expression	52 (24.1%)	37 (17.1%)	89 (41.2%)
Total	95 (44%)	121 (55.9%)	216 (100%)
			*P* < 0.01

χ^2^ Test was used to test the correlation between E2A and LGR5 expression (*P* < 0.01)

### E2A suppresses the tumor-initiating capacity of CRC cells in vitro

**Fig. 2:**
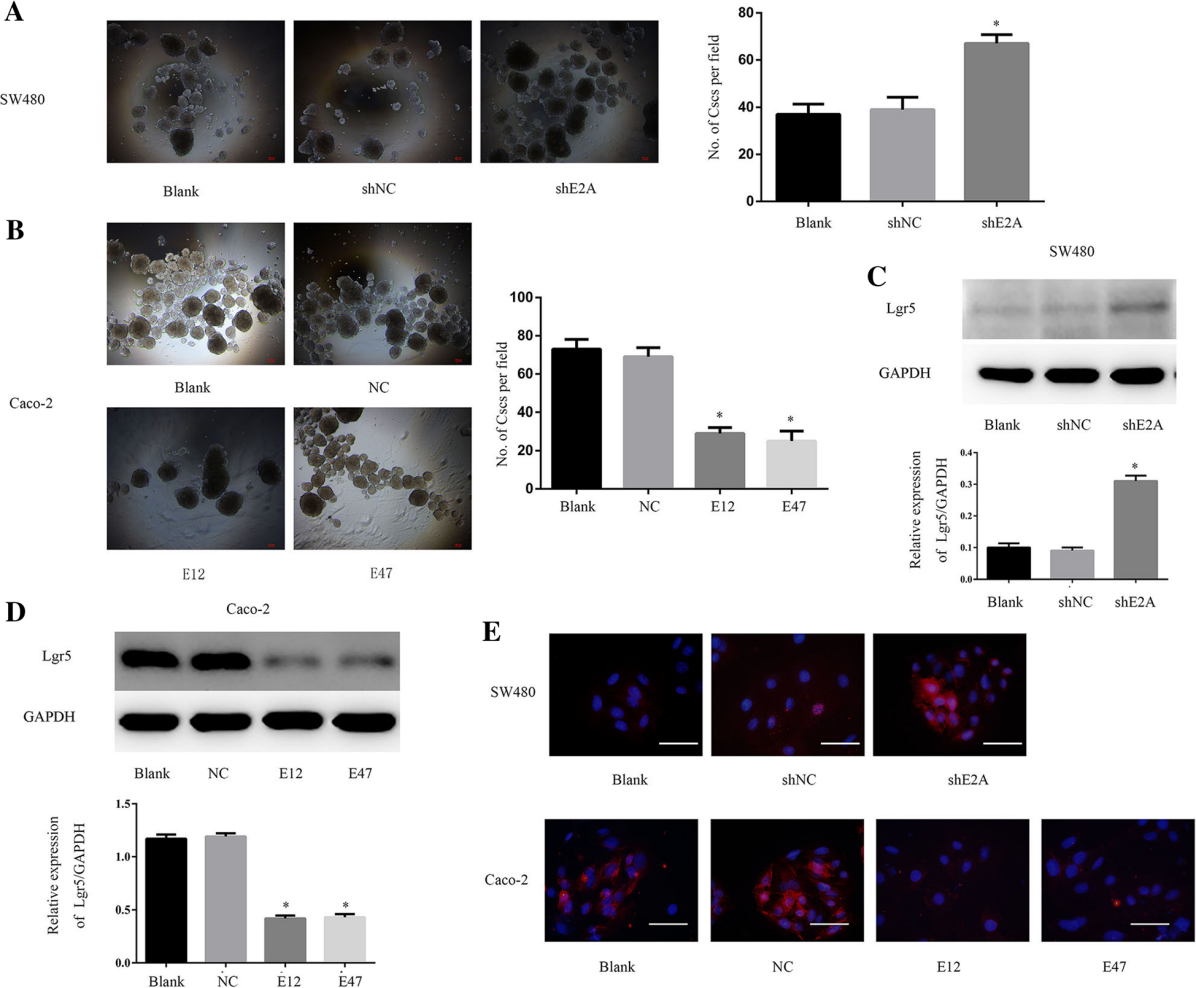
E2A suppressed the tumor-initiating capacity of CRC cells in vitro. **a** Effect of shE2A on SW480 cell sphere-formation ability. The sphere-formation ability of SW480 cells was enhanced by shE2A. *P* < 0.05, Scale bars: 50 μm. **b** Transfection of plasmids pEZ-M29-E12 and pEZ-M29-E47 to Caco-2 cells decreased the number of spheres per field. Left panel: Representative images of sphere formation assay are shown with original magnification of 40×. Scale bars: 50 μm. Right panel: Data in the histograms is expressed as means ± SD from three separate experiments. **c** Knockdown of E2A induced Lgr5 expression. Left panel: Immunoblot analysis showed that cancer stem-cell marker Lgr5 was increased in the shE2A-expressing SW480 clone, indicating induction of tumor-initiating ability. GAPDH was used as loading control. Right panel: Densitometric analysis of left panel normalized to GAPDH. **d** E12 and E47 suppressed Lgr5 expression. Left panel: Immunoblot analysis of Lgr5 and GAPDH expression with or without transfection of E12 or E47. Right panel: Densitometric analysis of left normalized to GAPDH. Data in the histograms is expressed as the means ± SD from three separate experiments. **e** Immunofluorescence analysis with anti-Lgr5 antibodies as indicated. Nuclei were counterstained with 4′, 6-diamidino-2-phenylindole (DAPI). Magnification: 400×; Scale: 50 μm. *, *P* < 0.05

We transfected LV-shE2A into SW480 cells to establish SW480/shE2A-stable clones, with LV-shNC as the control. In sphere-forming assays, SW480/shE2A cells had more tumor-initiating capacity than SW480 and SW480/shNC cells (Fig. 2a). On the other hand, when we transfected plasmid pEZ-M29-E12 or pEZ-M29-E47 into Caco-2 cells, to ectopically express E12 or E47 (named Caco-2/E12 and Caco-2/E47), both E12 and E47 reduced the sphere-forming capacity of the Caco-2 cells (Fig. 2b); there was no significant difference between Caco-2/E12 and Caco-2/E47 cells in this activity.

Given that Lgr5 is a crucial marker of CRC stem cells, we used western blot analysis to determine whether E2A regulates Lgr5 expression in CRC cells. As expected, Lgr5 expression in SW480 cells was increased after transfection with shE2A (Fig. 2c). Consistent with this result, Caco-2 cells transfected with E12 or E47 plasmid had decreased Lgr5 expression compared with that in Caco-2/NC cells (Fig. 2d). To further demonstrate the role of E2A in the regulation of Lgr5 expression, we performed immunofluorescence experiments to visualize Lgr5 in transfected SW480 cells. Consistent with immunoblot results, immunofluorescence showed that Lgr5 was increased in SW480/shE2A cells compared with that in SW480 and SW480/shNC cells. Furthermore, both E12 and E47 down-regulated the expression of Lgr5 in Caco-2 cells (Fig. 2e). Together, these results indicate that E2A can inhibit CRC cell tumor-initiating capacity.

### E2A suppressed CRC liver metastasis in vivo

**Fig. 3:**
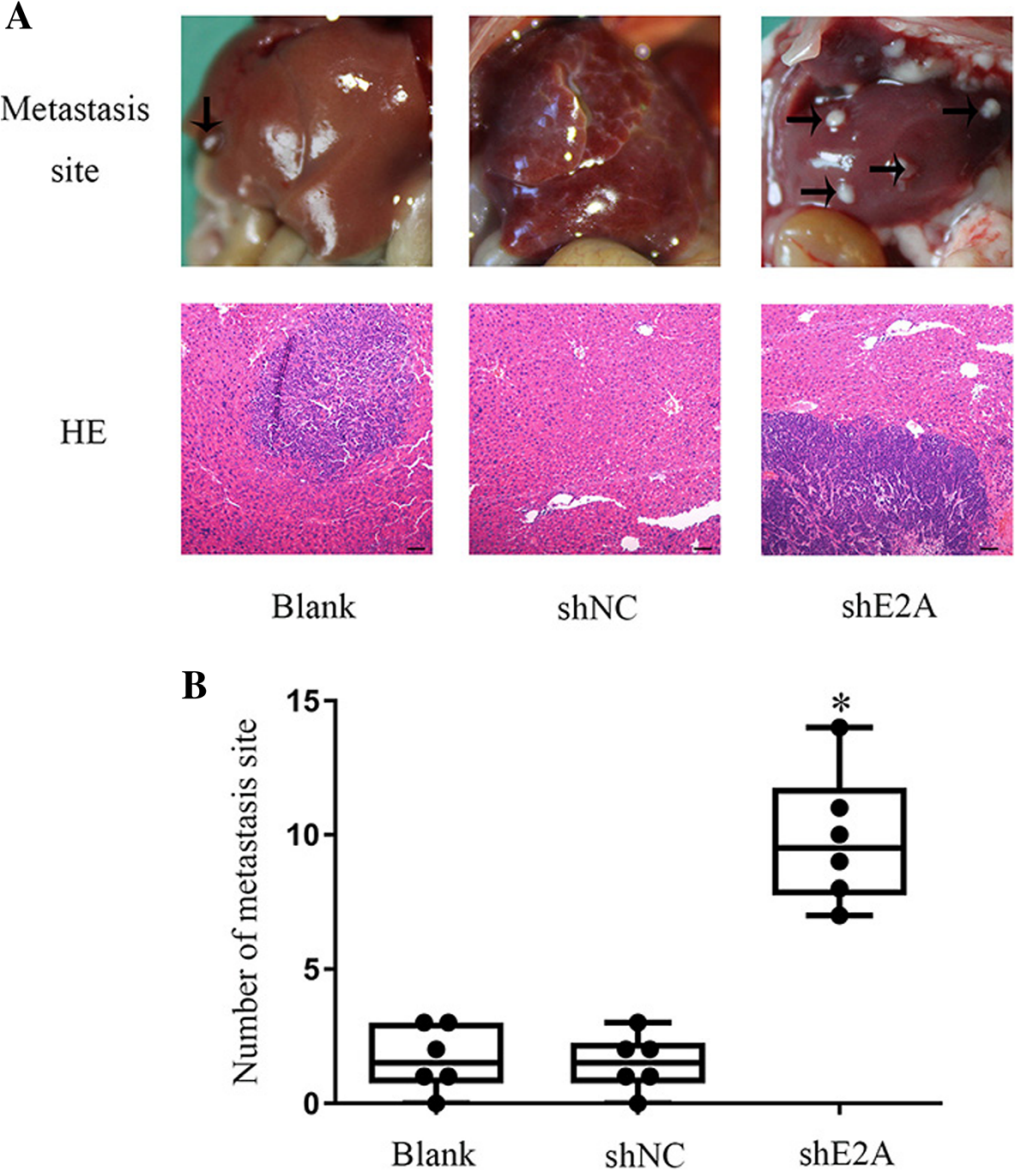
E2A suppress CRC liver metastasis in vivo. **a** Representative images of liver metastasis in SW480 cells in vivo. Scale bar: 100 μm. Magnification: 40×. Six mice are included in each group; (**b**) The number of metastatic nodules in each group. Data are presented as mean ± SD. *, *P* < 0.05

To determine whether E2A is involved in CRC cell tumor-initiating capacity in vivo, we generated a liver metastasis model with nude mice. Significantly more metastatic nodules developed in the SW480/shE2A mice than in the SW480 and SW480/shNC mice (Fig. 3a and b; *P* < 0.05). Evidently, CRC cells traveled to the liver through the portal vein, where their tumor-initiating ability induced proliferation.

### The Wnt/β-catenin pathway is crucial for E2A in CRC-cell tumor initiation

**Fig. 4:**
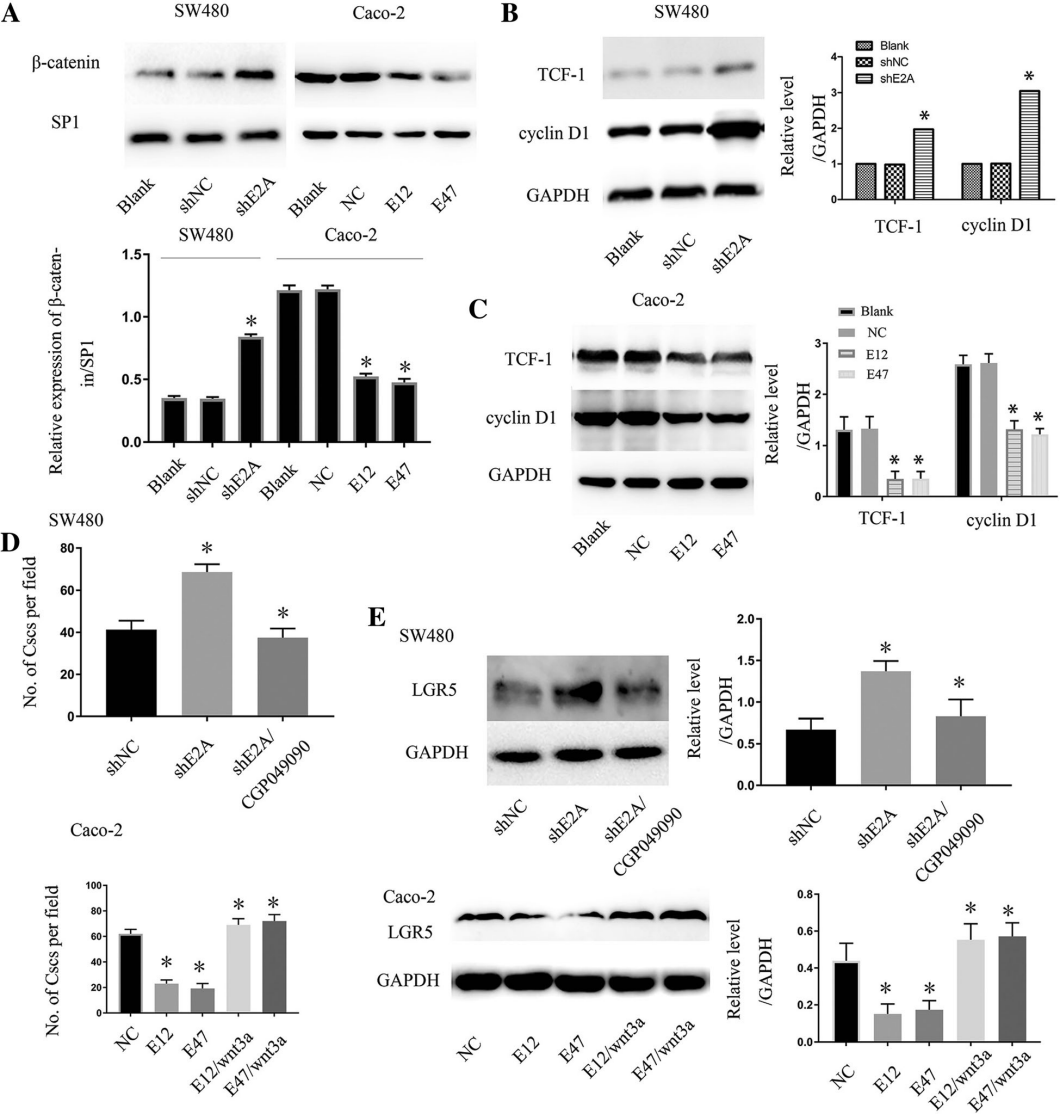
Wnt/β-catenin pathway is crucial for E2A in CRC cell tumor-initiating capacity. **a** Effect of E2A on the expression of β-catenin in cell nuclei. shE2A expression enhanced the β-catenin expression in SW480 cell nuclei, and E12/E47 attenuated the β-catenin expression in Caco-2 cell nuclei. Lower panel: Densitometric analysis of beta-catenin normalized to SP1. Data in the histograms is expressed as means ± SD from three separate experiments. **b**, **c** E2A suppressed the Wnt pathway in CRC cells. shE2A increased expression of the Wnt pathway effectors TCF-1 and cyclin D1 in SW480 cells, whereas E12 and E47 had the opposite effect in Caco-2 cells. Right panel: Densitometric analysis of TCF-1 and cyclin D1 normalized to GAPDH. Data in the histograms is expressed as mean ± SD from three separate experiments. **d** Wnt pathway is crucial for E2A suppressing tumor-initiating ability. SW480/shE2A cells treated with 5 μM CGP049090, which is a small-molecular compound inhibiting Wnt/β-catenin pathway activity, had decreased tumor-initiating ability compared with that of SW480/shE2A without CGP049090 (upper panel). wnt3a, at 100 ng/mL, added Caco-2 restored the tumor-initiating ability inhibited by E12/E47 to a level like that in Caco-2/NC cells (lower panel). **e** Lgr5 expression in SW480/shE2A cells treated with 5 μM CGP049090 was like that in SW480/shNC, which was lower than that in SW480/shE2A without CGP049090. wnt3a, at 100 ng/mL, added to Caco-2 cells, restored the Lgr5 expression inhibited by E12/E47 to a level like that in Caco-2/NC cells (lower panel). Data are the mean ± SD from at least three separate experiments. *, *P* < 0.05

Previously, we found that β-catenin was increased in SW480 cell nuclei after knockdown of E2A [24]. To confirm that observation, we performed nuclear protein extraction and western blot analyses. Figure 4a shows that β-catenin expression in SW480/shE2A cell nuclei was higher than in SW480/shNC and SW480 cell nuclei. In accordance with that finding, ectopic expression of E12 and E47 in Caco-2 cells reduced the amount of β-catenin in cell nuclei. To assess the influence of E2A on the Wnt/β-catenin pathway, we measured TCF-1 and cyclin D1 by Western blot, which are important effectors in the Wnt/β-catenin pathway [26]. As illustrated in Fig. 4b, the expression of TCF-1 and cyclin D1 were up-regulated by shE2A transfection. In Caco-2 cells, TCF-1 and cyclin D1 were decreased after E12 and E47 overexpression (Fig. 4c). We then investigated whether the decreased Wnt/β-catenin pathway was responsible for the E2A-induced inhibition of tumor-initiating capacity. SW480/shE2A cells were treated with CGP049090, which is a molecular inhibitor of the Wnt/β-catenin pathway. As illustrated in the upper panel of Fig. 4d, SW480/shE2A cells treated with CGP049090 had significantly less tumor-initiating capacity than did untreated cells, and the capacity was like that of SW480/shNC cells, as shown in sphere-formation assay. In addition, as illustrated in the upper panel of Fig. 4e, the same kind of change was seen with Lgr5: CGP049090 reduced the expression of Lgr5 in SW480/shE2A cells to the same level as in SW480/shNC cells. Next, we applied recombinant human Wnt3a, which can activate the Wnt/β-catenin pathway, to Caco-2/E12 and Caco-2/E47 cells to antagonize the effect of E12 and E47 on Caco-2 cells. As shown in Fig. 4d, Wnt3a enhanced the tumor-initiating capacity of Caco-2/E12 and Caco-2/E47 cells to the levels of Caco-2 cells. Also, the expression of Lgr5 was up-regulated after Caco-2/E12 and Caco-2/E47 cells were treated with Wnt3a (Fig. 4e, lower panel). Together, these experiments indicate that E2A impaired CRC cell tumor-initiating capacity in a Wnt/β-catenin-dependent manner.

### FoxM1 is a downstream target through which E2A suppresses CRC cell tumor-initiating capacity

**Fig. 5:**
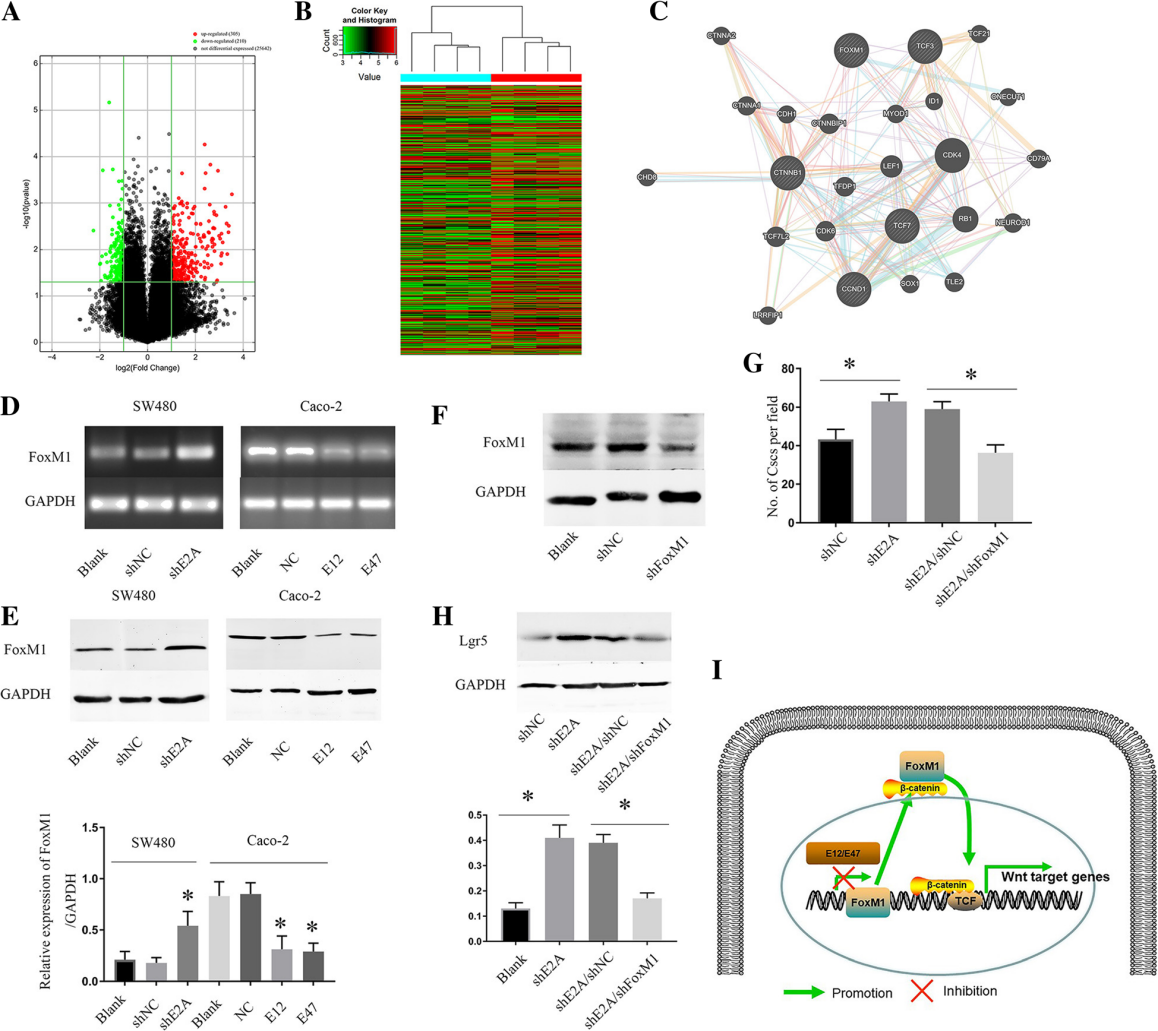
Foxm1 is a downstream target through which E2A suppressed CRC cell tumor-initiating capacity. **a**, **b** Heat map and volcano plot displaying the hierarchical clustering of the mRNA expression profiles. **c** PPI protein network generated by Genemania showing the relationship between E2A, FoxM1 and β-catenin. **d** Downregulation of E2A by shE2A resulted in increased FoxM1 mRNA expression in SW480 cells, whereas transfection of plasmids encoding E12 or E47 decreased FoxM1 mRNA in Caco-2 cells, as revealed by semi-qRT-PCR. **e** E2A decreased FoxM1 protein expression, as determined by immunoblot analysis. Lower panel: Densitometric analysis of FoxM1 normalized to GAPDH. Data in the histograms is expressed as the mean ± SD from three separate experiments. **f** Immunoblot analysis of FoxM1, with GAPDH as loading control. **g** shFoxM1 decreased tumor-initiating ability of SW480/shE2A cells to a level like that of SW480/shE2A cells. **h** Lgr5 expression in SW480/shE2A cells treated with shFoxM1 is like that in SW480/shNC, which is lower than in SW480/shE2A. Lower panel: Densitometric analysis of Lgr5 normalized to GAPDH. Data in the histograms is expressed as mean ± SD from three separate experiments. **i** Schematic diagram illustrates that E2A inhibited FoxM1 transcription to attenuate FoxM1, depending on β-catenin translocation to cell nuclei, which decreased the Wnt pathway in CRC cells

To further identify new targets of E2A and understand the mechanisms underlying E2A’s tumor suppressive function in CRC, we used mRNA microarray for a target search (Fig. 5a and b). Among the genes, FoxM1 had different effects on SW480/shE2A and SW480/shNC cells. Furthermore, previous reports indicated that FoxM1 promotes β-catenin nuclear localization and regulates stemness of CRC cells [27, 28]. According to the UALCAN database (http://ualcan.path.uab.edu/index.html, accessed 24 May 2018), FoxM1 expression was higher in colon cancer tissues than in normal tissues (Additional file 1: Figure S1 A, *P* < 0.05). Furthermore, Kaplan-Meier survival curves revealed that high expression of FoxM1 in colon cancer correlated with poor survival (Additional file 1: Figure S1 B, *P* < 0.05). Additionally, the PPI (Protein-protein interaction) protein network generated by Genemania (http://genemania.org/, accessed 18 July 2018) showed the relationship between E2A, FoxM1 and β-catenin (Fig. 5c). To validate the change in expression of FoxM1, we performed RT-PCR of SW480 and Caco-2 cells and found that FoxM1 mRNA was increased in SW480/shE2A cells; however, it was decreased when we transfected E12 and E47 into Caco-2 cells (Fig. 5d). In parallel with the mRNA alteration, the FoxM1 protein expression level of SW480 cells was up-regulated by shE2A and decreased in Caco-2 cells by E12 and E47 plasmid transfection (Fig. 5e). Together, these experiments indicate that FoxM1 mRNA and protein levels are regulated by E2A.

Next, we investigated whether the increased FoxM1 in SW480/shE2A cells can lead to enhanced tumor-initiating capacity. To this end, transient transfection of shFoxM1 was performed in SW480/shE2A cells to down-regulate FoxM1 (Fig. 5f). As illustrated in Fig. 5g, compared with cells transfected with negative control or blank cells, the sphere-forming ability of SW480/shE2A cells was reduced by shFoxM1 to the same levels as that in SW480/shNC cells. This finding suggests that the enhanced FoxM1 by shE2A in SW480 cells is critical in the regulation of sphere formation. Besides, downregulation of FoxM1 decreased the expression of Lgr5 in SW480 cells (Fig. 5h). More importantly, β-catenin expression in Caco-2 cell nuclei was reduced to 40% of normal values after shFoxM1 transfection, and changes of TCF-1 and cyclin D1 suggested there was suppression of the Wnt/β-catenin pathway (Additional file 1: Figure S1D). To further define the role of FoxM1 in β-catenin regulation in cell nuclei, we performed immunofluorescence experiments to visualize β-catenin in transfected Caco-2 cells. In accordance with immunoblot results, these experiments revealed that β-catenin was decreased in Caco-2 cell nuclei after shFoxM1 transfection (Additional file 1: Figure S1C). Thus, FoxM1 is a target through which E2A regulates the Wnt/β-catenin pathway to suppress invasion and migration in CRC cells. A schematic diagram of our study results is presented in Fig. 5i.

## Discussion

Distant metastases, rather than the primary tumors from which they arise, are responsible for > 90% of carcinoma-associated mortality [29]. The formation of metastatic tumors is a multistep process, mediated by complex cascades of molecular events that govern cancer-cell migration and tumor-initiating ability to induce cancer-cell proliferation in distant organs [30]. In our previous study, E2A inhibited the invasion/migration process by reducing YAP expression in CRC cells, in which β-catenin translocation to cell nuclei occurred after silencing E2A expression in SW480 cells [24]. As a core factor in canonical Wnt signaling, β-catenin translocated to cell nuclei, bound to transcriptional complexes, and promoted transcription of targeting genes, such as cyclin D1 and TCF1 [31]. Given that the canonical Wnt pathway activates the stem-cell property of cancer cells, in the present study, we demonstrated the suppressive role of E2A in CRC cell tumor-initiating capacity by regulating β-catenin translocation. Furthermore, we found that FoxM1 is a downstream target of E2A in CRC cells.

E2A has been called a regulator of early B-cell development, and it is dysregulated in various cancers [32–34]. Zhu et al. [35] found that PAK-5-mediated E47 phosphorylation promotes EMT, and we found that E2A inhibited EMT in a YAP-dependent manner [24]; this was an intriguing finding. The explanation for this observation may be that E47 participates in CRC cell progression via various pathways; more research is needed to elucidate the various roles of E2A in CRC cells. To clarify the correlation between E2A expression and progression-free survival in CRC patients, we validated E2A as a prognostic factor in 216 CRC patients. The reverse correlation between E2A and Lgr5 expression in CRC tissues gave us a clue for further research.

To identify the influence of E2A on tumor-initiating ability, we performed sphere- formation assays, finding that E2A decreased the ability of CRC cells to form spheres. Lgr5, a seven-transmembrane protein of the class A rhodopsin-like family of G protein-coupled receptors, is a stem-cell marker in intestinal cells. Lgr5+ cells divide every 24 h, generating rapidly proliferative progenitors that fill the pocket-like crypts [36, 37]. It has been reported that stem cell/progenitor hierarchies are maintained in CRC tissue and that LGR5 is a cancer stem-cell marker [38]. Consistent with results of the sphere-formation assay, we found that E2A inhibited expression of Lgr5 in Caco-2 cells and silenced up-regulated E2A expression of Lgr5 in SW480 cells. To this end, we documented that E2A participated in regulation of tumor-initiating capacity. In our previous study [24], inhibition of E2A expression in CRC cells resulted in overexpression of β-catenin, and immunofluorescence analysis showed that the increased β-catenin was not located on the cytomembrane, but in cell nuclei. To verify β-catenin overexpression in CRC cell nuclei, we extracted nuclear protein and measured it in western blot experiments. As expected, β-catenin accumulated in cell nuclei after E2A silencing. Corresponding with that observation, expression of TCF-1 and cyclin D1 was diminished after E12 and E47 overexpression. We affirmed that E2A exerts its suppressive function of the Wnt pathway by inhibiting β-catenin accumulation in CRC cell nuclei.

Forkhead box M1 (FoxM1) is one of the members of the Forkhead family that are conserved transcriptional regulators, which are defined by a common DNA-binding domain that is termed the Forkhead box [39]. FoxM1 is involved in cell proliferation and apoptosis, thus affecting the development of many organs [40]. FoxM1 is overexpressed in various human cancers, and it has been implicated in all major hallmarks of cancer, such as metastasis and chemoresistance [28, 41–43]. In glioma, Zhang et al. [28] demonstrated that β-catenin translocation to nuclei depends on its binding to FoxM1, which drives the formation of gliomas [27]. In this study, we documented that E2A suppresses the expression of FoxM1 at the mRNA and protein levels, and we demonstrated that E2A influences tumor-initiating ability in a FoxM1-dependent manner. In CRC cells, FoxM1 participated in β-catenin translocation to nuclei and activation of Wnt signaling, a finding that is consistent with the work of Zhang et al. [28] in gliomas. Considering that total β-catenin expression decreased in CRC cells after E2A overexpression and that FoxM1 regulated the translocation of β-catenin, more study is needed to clarify the mechanism of E2A regulating β-catenin expression in CRC cells; this will be emphasized in our future work. Nonetheless, in this study, we demonstrated that FoxM1 is a downstream target in mediating E2A’s function as a tumor suppressor gene in CRC.

## Conclusion

The findings of this study established that E2A expression is associated with CRC cell tumor-initiating ability. By targeting FoxM1, E2A inhibits β-catenin in cell nuclei and suppresses the tumor-initiating capacity of CRC cells. Although the function of E2A in cancer has not been fully defined, we identified a new molecular target and mechanism of action of E2A in CRC. E2A has the potential to be a target for the prevention and therapy of CRC.

## Additional files

Additional file 1: **Figure S1.** (A) FoxM1 expression was higher in colon cancer tissues than in normal tissues, which is generated from UALCAN database. (B) According to UALCAN database, the Kaplan-Meier survival curve demonstrates high expression of FoxM1 in colon cancer correlated with poor survival. (C) shFoxM1 decreased β-catenin translocation to cell nuclei in Caco-2 cells, as immunofluorescence analysis shows. Nuclei were counterstained with DAPI. Magnification: 400×; Scale: 50 μm. (D) shFoxM1 decreased β-catenin in Caco-2 cell nuclei, as revealed by immunoblot analysis, with SP1 as loading control. TCF-1 and cyclin D1 expression was inhibited by shFoxM1. Right panel: Densitometric analysis of left normalized to GAPDH. *, *P* < 0.05. (JPG 261 kb)

Additional file 2: **Figure S2.** (A) E2A increased phospho-β-catenin protein expression, as determined by immunoblot analysis. Right panel: Densitometric analysis of phospho-β-catenin normalized to GAPDH. Data in the histograms are expressed as the mean ± SD from three separate experiments. (B) TCF/LEF reporter luciferase assay was used as a reporter for the determination of Wnt/β-catenin pathway activity. ShE2A increased the Wnt/β-catenin pathway activity, whereas CGP049090 attenuated the activity. Data in the histograms are expressed as the mean ± SD from three separate experiments. *, *P* < 0.05. (JPG 517 kb)

## Data Availability

All data generated or analysed during this study are included in this published article and its Additional files 1 and 2.

## Abbreviations

bHLH: basic helix–loop–helix
CRC: Colorectal cancer
EMT: Epithelial-mesenchymal transition
FoxM1: Forkhead box M1
GAPDH: Glyceraldehyde-3-phosphate dehydrogenase
HRP: Horseradish peroxidase
shNC: shRNA-negative control
shRNA: Short hairpin RNA
SP1: Specificity protein 1
