# Supplementary figures and images for: E2A attenuates tumor-initiating capacity of colorectal cancer cells via the Wnt/beta-catenin pathway
Source: J Exp Clin Cancer Res. 2019 Jun 24;38:276. doi: 10.1186/s13046-019-1261-5 (PMC6591938; doi:10.1186/s13046-019-1261-5)

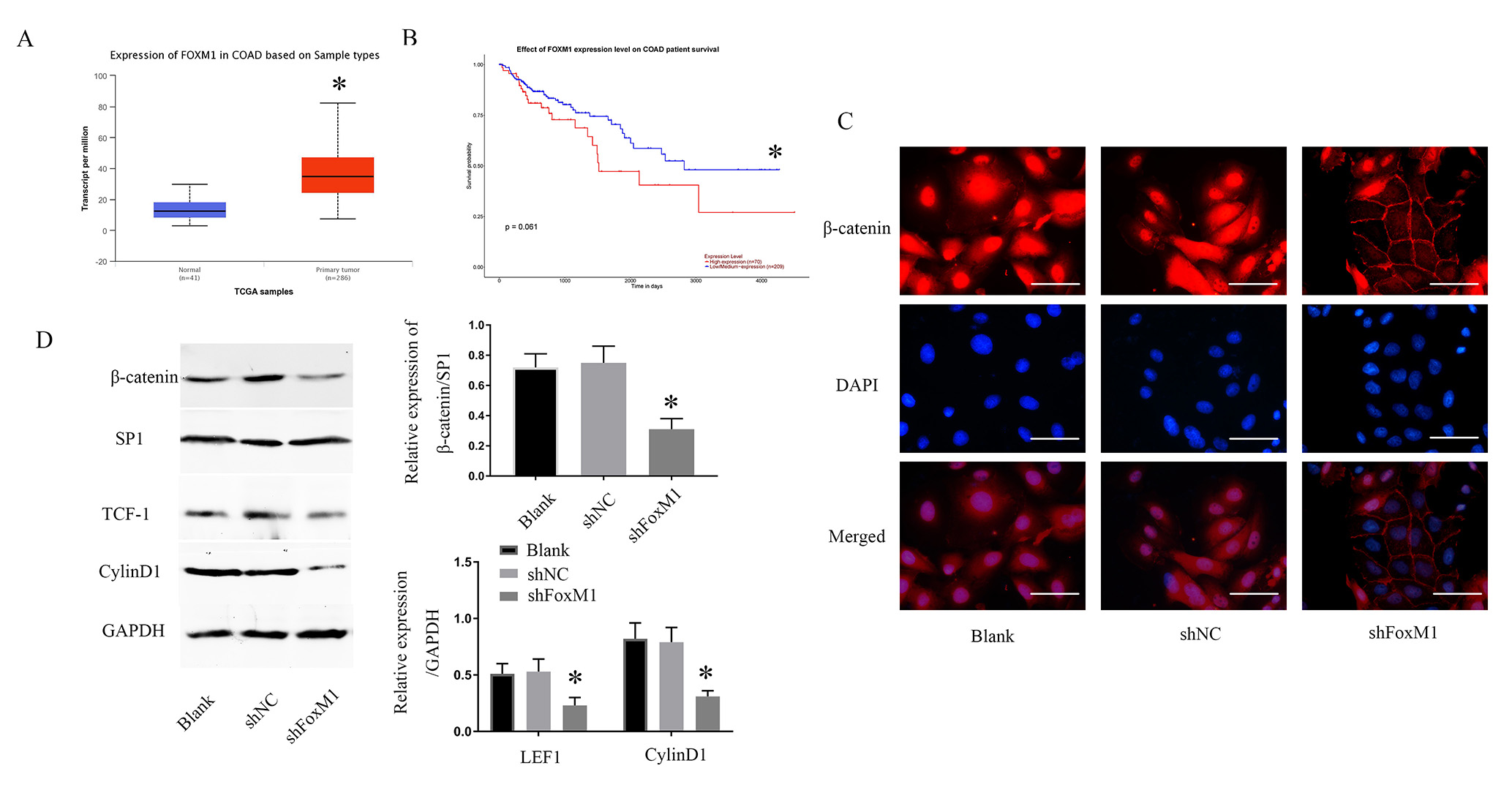

Supplement: Supplementary file 1 — Figure S1. (A) FoxM1 expression was higher in colon cancer tissues than in normal tissues, which is generated from UALCAN database. (B) According to UALCAN database, the Kaplan-Meier survival curve demonstrates high expression of FoxM1 in colon cancer correlated with poor survival. (C) shFoxM1 decreased β-catenin translocation to cell nuclei in Caco-2 cells, as immunofluorescence analysis shows. Nuclei were counterstained with DAPI. Magnification: 400×; Scale: 50 μm. (D) shFoxM1 decreased β-catenin in Caco-2 cell nuclei, as revealed by immunoblot analysis, with SP1 as loading control. TCF-1 and cyclin D1 expression was inhibited by shFoxM1. Right panel: Densitometric analysis of left normalized to GAPDH. *, P < 0.05. (JPG 261 kb) [file 13046_2019_1261_MOESM1_ESM.jpg]

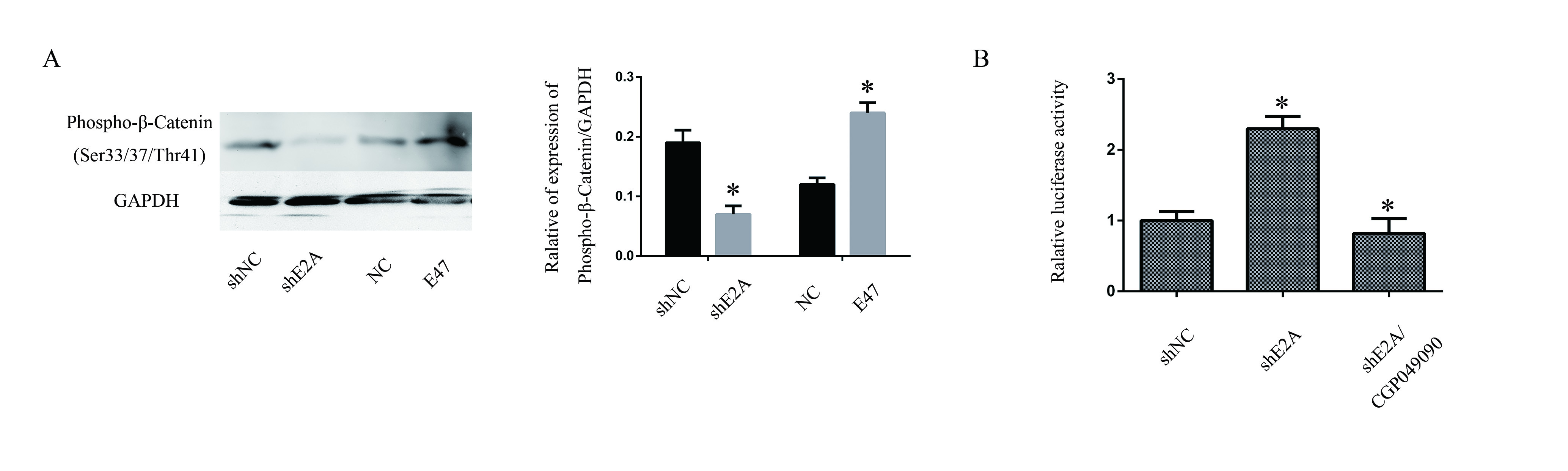

Supplement: Supplementary file 2 — Figure S2. (A) E2A increased phospho-β-catenin protein expression, as determined by immunoblot analysis. Right panel: Densitometric analysis of phospho-β-catenin normalized to GAPDH. Data in the histograms are expressed as the mean ± SD from three separate experiments. (B) TCF/LEF reporter luciferase assay was used as a reporter for the determination of Wnt/β-catenin pathway activity. ShE2A increased the Wnt/β-catenin pathway activity, whereas CGP049090 attenuated the activity. Data in the histograms are expressed as the mean ± SD from three separate experiments. *, P < 0.05. (JPG 517 kb) [file 13046_2019_1261_MOESM2_ESM.jpg]
